# Case Report: Prenatal diagnosis of a rare complex fetal karyotype 47,U,t(10;13)(p15;q22)mat,+der(13)t(10;13)dmat resulting from 3:1 meiotic segregation of a maternal balanced translocation

**DOI:** 10.3389/frph.2025.1737392

**Published:** 2025-12-17

**Authors:** G. S. Deng, D. H. Zhang, Y. Q. Lai, J. J. Song, J. J. Pan, X. F. Liang, Y. H. Lu, S. S. Ning, W. C. Li, X. Li, Y. Y. Chen, D. R. Li, L. L. Li, Y. N. Liang

**Affiliations:** Yulin Women and Children Health Care Hospital, Yulin, Guangxi, China

**Keywords:** balanced translocation, karyotype analysis, CNV-seq, CCRs, sSMC, PGT

## Abstract

**Objective:**

To characterize a rare fetal complex chromosomal rearrangement (CCR) derived from a maternal balanced translocation using integrated G-banding and CNV-seq analysis.

**Methods:**

Integrated G-banding and CNV-seq enabled precise karyotypic determination in the fetus, with familial verification confirming its derivation.

**Results:**

Karyotype analysis confirmed that the pregnant woman was a carrier of a balanced translocation, 46,XX,t(10;13)(p15;q22), while her husband had a normal karyotype. Combined G-banding and CNV-seq analyses diagnosed the fetal karyotype as 47,U,t(10;13)(p15;q22)mat,+der(13)t(10;13)dmat, resulting from 3:1 meiotic segregation of the maternal balanced translocation.

**Conclusion:**

This case confirms the pivotal role of integrated G-banding and CNV-seq in diagnosing complex chromosomal rearrangements. For families with a high recurrence risk, PGT is a mandatory intervention to prevent subsequent adverse reproductive outcomes.

## 1 Introduction

Reciprocal translocation (t) is a structural rearrangement caused by the exchange of segments between two chromosomes (1). Balanced translocations have an estimated prevalence of 0.2% in the general population and represent a major cause of recurrent pregnancy loss, early embryonic demise, and congenital anomalies in offspring (2, 3). This occurs due to erroneous meiotic segregation, which generates unbalanced gametes. Consequently, embryos may develop with aneuploidy or segmental aneusomy, thereby explaining the adverse pregnancy outcomes (4). During meiotic metaphase, the translocated chromosomes and their normal homologs can form a quadrivalent structure. The segregation of this structure (e.g., 2:2, 3:1) can theoretically yield a spectrum of at least 18 different gametic types, of which only two result in a normal or balanced karyotype (5). In practice, the array of possible gametes is even more complex, and their production occurs with unequal probability. Consequently, accurately predicting the proportion of offspring with a normal or balanced karyotype poses a significant challenge (6). Combining G-banding and CNV-seq, this study analyzed a fetus with cardiac defects, bilateral choroid plexus cysts, and high-risk signs of trisomy 18 to trace a balanced chromosomal translocation to its maternal origin and examine the mechanisms of gamete formation. The results offer valuable evidence for guiding genetic counseling and prenatal diagnosis for such disorders.

## 2 Patients and methods

### 2.1 Clinical history

The patient was a 22-year-old female, gravida 5 para 2 (with one healthy daughter and one healthy son), with a history of two induced abortions. The current pregnancy was spontaneously conceived. First-trimester serum screening at 13 weeks indicated a high risk for trisomy 18. A follow-up ultrasound at 17 weeks revealed fetal cardiac structural abnormalities, suggestive of double outlet right ventricle (DORV), accompanied by bilateral choroid plexus cysts. Amniocentesis was performed at 19^+^ weeks. Initial G-banding karyotyping of the fetus indicated 47,U,t(10;13)(p15;q22),+?der(13)t(10;13). Parental karyotyping identified a balanced translocation 46,XX,t(10;13)(p15;q22) in the mother and a normal male karyotype (46,XY) in the father. CNV-seq on the fetal sample identified a 3.90 Mb duplication at 10p15.3-p15.1 and a 55.72 Mb duplication at 13q11-q22.1. Integrating the G-banding and CNV-seq findings, the final fetal karyotype was confirmed as 47,U,t(10;13)(p15;q22)mat,+der(13)t(10;13)dmat, resulting in partial trisomy 10p and partial trisomy 13q of maternal origin. Following genetic counseling, pregnancy termination was elected. A stillborn female fetus was delivered, with no notable dysmorphic features. The family history was non-contributory.

### 2.2 Methods

Following informed consent, amniotic fluid and parental peripheral blood samples were collected. Amniotic fluid samples were cultured using the monolayer method in 25 cm^2^ culture flasks. Following centrifugation, the cell suspension was inoculated into flasks and cultured until a confluent monolayer formed. Parental blood lymphocytes were cultured with phytohemagglutinin (PHA) stimulation. G-banded karyotypic analysis was performed following standard cytogenetic protocols, with 30 metaphase spreads examined and 5 karyotypes analyzed per sample. Chromosomal nomenclature adhered to ISCN 2024 (7). Genomic DNA was extracted from the samples using a commercial kit (Beijing BerryGenomics Co., Ltd.). Library construction and sequencing were performed on the NextSeq CN500 platform. The resulting sequencing data were aligned to the human reference genome (hg19) for coverage depth calculation and the identification of chromosomal abnormalities. Detected copy number variations were annotated by referencing public genomic databases, including OMIM, DECIPHER, DGV, and UCSC, supplemented by literature reviews via PubMed. Variant classification was conducted in accordance with ACMG guidelines.

## 3 Results

**Figure 1:**
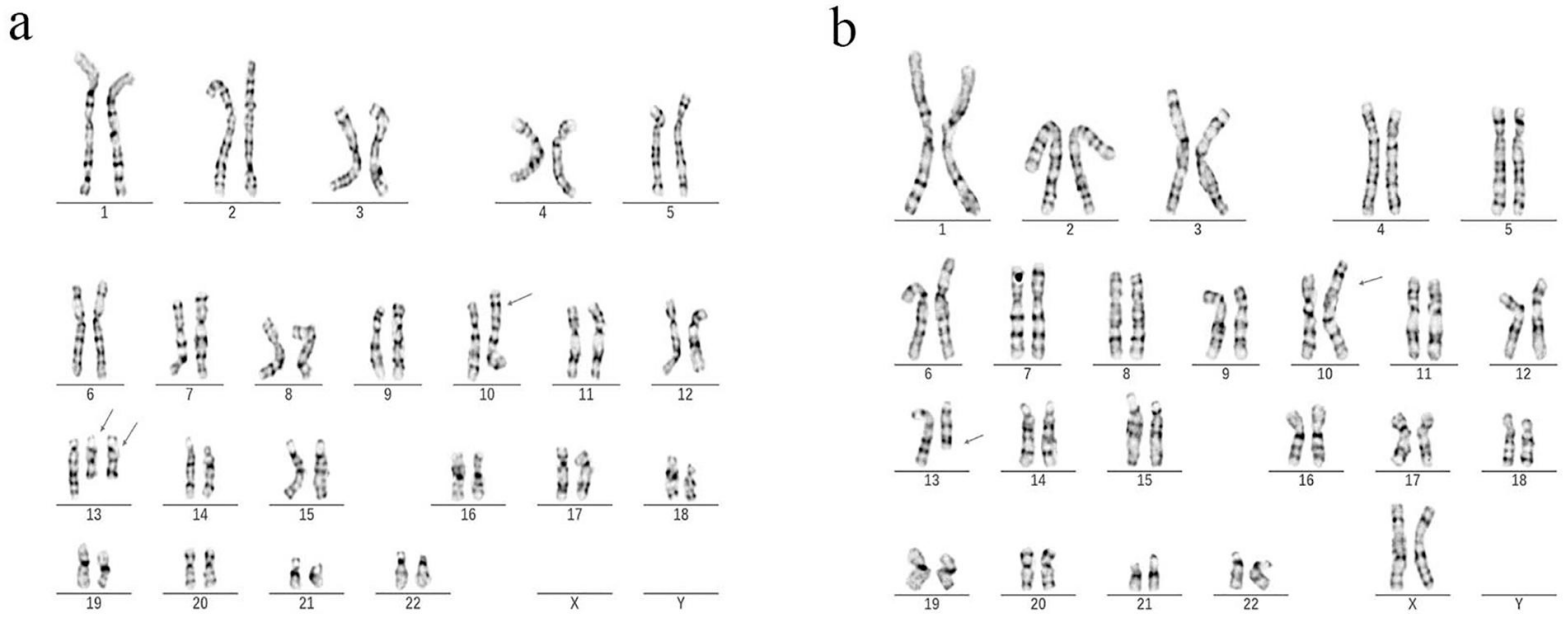
G-banded karyotype analysis. **(a)** Fetal karyotype showing 47,U,t(10;13)(p15;q22)mat,+der(13)t(10;13)dmat. **(b)** Maternal karyotype revealing a balanced translocation: 46,XX,t(10;13)(p15;q22).

**Figure 2:**
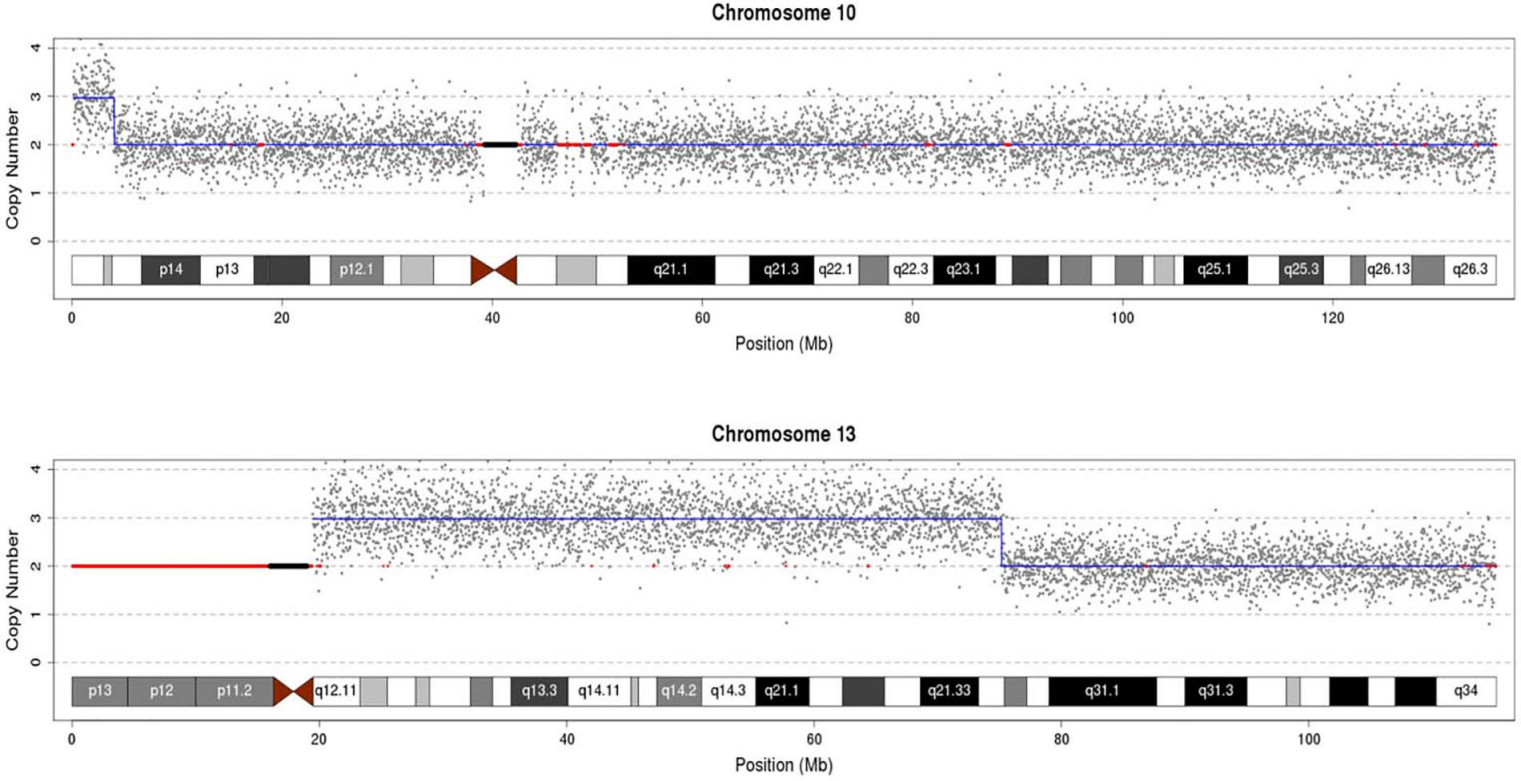
CNV-seq results from the fetal amniotic fluid sample.

G-banded cytogenetic analysis of the fetal amniotic fluid sample revealed an initial karyotype of 47,U,t(10;13)(p15;q22),+?der(13)t(10;13) (Figure 1a). Familial studies confirmed a balanced translocation, 46,XX,t(10;13)(p15;q22), in the mother (Figure 1b), while the father exhibited a normal male karyotype of 46,XY. CNV-seq analysis identified two chromosomal duplications in the fetus: an approximately 3.90 Mb duplication at 10p15.3-p15.1, designated as seq[hg19] dup(10)(p15.3p15.1) chr10:g.120000_4020000dup, and an approximately 55.72 Mb duplication at 13q11-q22.1, designated as seq[hg19] dup(13)(q11q22.1) chr13:g.19440000_75160000dup (Figure 2). Integrative analysis of G-banding and CNV-seq results confirmed that the extra der(13) chromosome originated from the maternal balanced translocation. The final karyotype was determined as 47,U,t(10;13)(p15;q22)mat,+der(13)t(10;13)dmat. This was interpreted as resulting from unbalanced segregation of the maternally derived balanced translocation, leading to partial trisomy 10p and partial trisomy 13q in the fetus.

## 4 Discussion and conclusion

**Figure 3:**
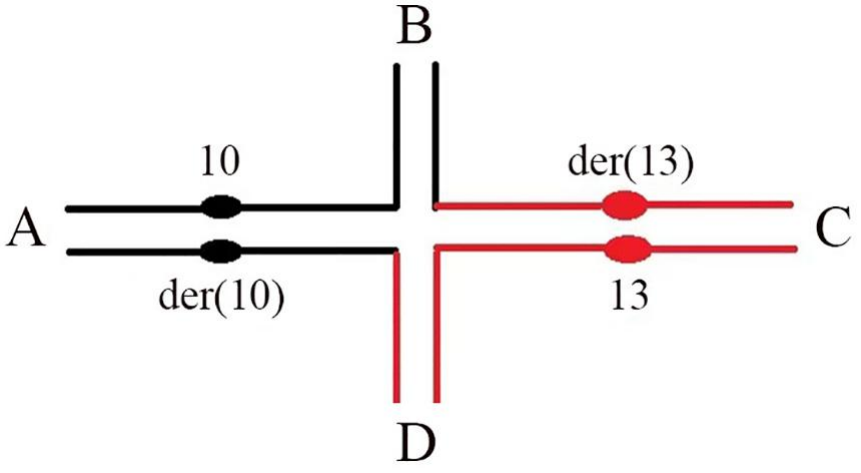
Schematic representation of the quadrivalent formed by homologous pairing of the translocated chromosomes in the mother.

Derivative chromosomes (der), which typically arise from structural rearrangements or complex abnormalities, are commonly found in the offspring of individuals carrying balanced translocations or inversions. This case presents a fetal karyotype characterized by a large supernumerary derivative chromosome 13 [+der(13)t(10;13)] originating from a parental balanced translocation. Although its formation mechanism—specifically, the 3:1 unbalanced segregation of t(10;13) producing + der(13)—is analogous in principle to that of the well-established complex sSMC model seen in Emanuel syndrome [derived from t(11;22)] (8), our case exhibits distinct characteristics in terms of the translocation type, the specific composition of the marker chromosome, and its exceptionally large size (with a 55.72 Mb duplication of 13q). This resulted in the fetus simultaneously bearing large partial trisomies of 10p and 13q, leading to a composite phenotype that superimposes the known manifestations of 13q duplication syndrome onto neurodevelopmental abnormalities associated with 10p duplication (9). Compared to over 150 complex sSMC cases documented in the global sSMC database (10), the present case aligns with established genotype-phenotype correlations: most reported cases share common clinical features such as intellectual disability and multiple malformations, with phenotypic severity being closely related to the size, gene content, and parental origin of the sSMC (11, 12). It is particularly noteworthy that this study may represent one of the first reported cases worldwide to fully elucidate the origin of a large supernumerary derivative chromosome 13 [+der(13)] resulting from 3:1 segregation of a clearly defined maternal t(10;13) balanced translocation. This finding not only expands the spectrum of large marker chromosomes derived from balanced translocations but also provides important new evidence for understanding the genotype-phenotype relationships in such CCRs. While phenotypically normal, carriers form a quadrivalent structure during meiosis (Figure 3) (13, 14), whose segregation through alternate, adjacent-1, adjacent-2, or 3:1 modes is classically thought to yield at least 18 gamete types (Table 1). Critically, the fetal karyotype we observed is not represented in this classical set. This discrepancy is addressed by ISCN 2024 (7), which indicates that considering crossovers between centromeres and breakpoints would add 8 more types. Expanding on this, Wang Hao et al. (15) integrated 2:2 (with odd/even crossovers), 3:1, and 4:0 segregation modes to propose a maximum of 36 theoretical gamete types. The karyotype described here is inferred to stem from a 3:1 segregation with an odd crossover—a rare combination even within the expanded model of 36 types.

**Table 1:** Theoretical gamete types resulting from meiotic segregation of maternal t(10;13)(p15;q22)^a^.

Segregation mode	Segregation diagram	Chromosomal constitution	Resulting zygote karyotype
Alternate (2:2)	AB CD	10, 13	46,XY
	AD CB	der(10), der(13)	46,XY,t(10;13)(p15;q22)mat
Adjacent-1 (2:2)	AB CB	10, der(13)	46,XY,der(13)t(10;13)(p15;q22)dmat
	AD CD	der(10), 13	46,XY,der(10)t(10;13)(p15;q22)dmat
Adjacent-2^b^ (2:2)	AB AD	10, der(10)	46,XY,+der(10)t(10;13)(p15;q22)dmat,−13
	CD CB	13, der(13)	46,XY,−10,+der(13)t(10;13)(p15;q22)dmat
	AB AB	10, 10	46,XY,+10,−13
	AD AD	der(10), der(10)	46,XY,der(10)t(10;13)(p15;q22)dmat,+der(10)t(10;13),−13
	CB CB	der(13), der(13)	46,XY,−10,der(13)t(10;13)(p15;q22)dmat,+der(13)t(10;13)
	CD CD	13, 13	46,XY,−10,+13
3:1 Segregation	AD CD CB	der(10), 13, der(13)	47,XY,t(10;13)(p15;q22)mat,+13
	AB	10	45,XY,−13
	AB CD CB	10, 13, der(13)	47,XY,+der(13)t(10;13)(p15;q22)dmat
	AD	der(10)	45,XY,der(10)t(10;13)(p15;q22)dmat,−13
	AB AD CD	10, der(10), 13	47,XY,+der(10)t(10;13)(p15;q22)dmat
	CB	der(13)	45,XY,−10,der(13)t(10;13)(p15;q22)dmat
	AB AD CB	10, der(10), der(13)	47,XY,+10,t(10;13)(p15;q22)mat
	CD	13	45,XY,−10

a Only gametes from alternate segregation (AB CD and AD CB) yield balanced chromosomal constitutions; all other types result in genomic imbalance.

b Adjacent-2 segregation is expected to yield at least the first two unbalanced gamete types listed in the table (AB AD and CD CB). The remaining four gamete types require a crossover between the centromere and the translocation breakpoint.

Traditional genetic counseling based on the “18-gamete model” often misestimates reproductive risks for balanced translocation carriers, as gamete probabilities are unequal. While 2:2 segregation is the predominant mode (16), the unusual fetal karyotype in this case is attributed to a rarer 3:1 segregation event. CNV-seq identified concurrent duplications at 13q11-q22.1 (55.72 Mb; 168 genes) and 10p15.3-p15.1 (3.90 Mb; 12 genes), associated with neurodevelopmental abnormalities including intellectual disability, microcephaly, and autism (17). These findings suggested a parental balanced translocation, which was confirmed by familial karyotyping as a maternal t(10;13)(p15;q22). The fetal karyotype was ultimately defined as resulting from unbalanced segregation of the maternal translocation, leading to partial trisomy 10p and 13q. Given the significantly elevated risk of unbalanced karyotypes in the offspring of balanced translocation carriers, prenatal diagnosis or preimplantation genetic testing (PGT) is strongly recommended in subsequent pregnancies to prevent the birth of children with chromosomal disorders.

In summary, the precise diagnosis of complex chromosomal rearrangements requires an integrated approach combining cytogenetic and molecular genetic techniques. In this case, the combined application of G-banding and CNV-seq precisely identified the rare fetal karyotype and established its origin from 3:1 meiotic segregation of a maternal balanced translocation, thereby providing crucial information for genetic counseling and prenatal diagnosis. Considering the high recurrence risk for this family, PGT is recommended as a vital option in future reproductive planning.

## Data Availability

The original contributions presented in the study are included in the article/Supplementary Material, further inquiries can be directed to the corresponding authors.
